# Profile of metabolic abnormalities seen in patients with type 2 diabetes mellitus and their first degree relatives with metabolic syndrome seen in Benin City, Edo state Nigeria

**DOI:** 10.1186/2251-6581-13-61

**Published:** 2014-05-23

**Authors:** Stephen O Ogedengbe, Ignatius U Ezeani

**Affiliations:** 1 Departments of Medicine, University of Benin Teaching Hospital, Benin City and Federal Medical Center, P.M.B 7001, Umuahia, Abia state, Nigeria

**Keywords:** Metabolic syndrome, Biochemical abnormalities, Type 2 diabetes mellitus, Anthropometry, Hypertension, Obesity, Dyslipidemia

## Abstract

**Background:**

To determine the profile of metabolic abnormalities in T2DM persons with metabolic syndrome and their non-diabetic first-degree relatives who also had metabolic syndrome in Benin City.

**Methodology:**

This was a cross sectional case controlled study in which convenience sampling technique was used to recruit 106 persons with T2DM, 96 people who are first degree relatives of type 2 diabetic persons and 96 controls using a interviewer administered questionnaire technique. The following were assessed: anthropometric indices, blood pressure, serum lipid profile, fasting blood sugar, proteinuria, and microalbuminuria. The data obtained were analyzed using the statistical software-Statistical package for social sciences [SPSS] version 16. A p-value of less than 0.05 was taken as statistically significant.

**Results:**

The mean age (SD) of the study groups were: persons living with T2DM: 58.6 ± 11.2 years, control: 57.69 ± 60.8 years and FDR: 57.4 ± 10.6 years. No significant age and sex differences were observed in these groups. There were more females (59.7%) than males (40.3%) with T2DM*.* The prevalence of MS was 13.5%, 16.7%, and 87.1% in the control, FDR and T2DM patients respectively. For the T2DM group of subjects, impaired fasting glycaemia was the commonest metabolic abnormality followed by microalbuminuria, low HDL cholesterol, high LDL cholesterol, hypercholesterolaemia and hypertriglyceridaemia in decreasing frequency. For the FDR group, low HDL cholesterol was the commonest metabolic abnormality followed by hypertriglyceridaemia, impaired fasting glucose, high LDL cholesterol, hypertriglyceridaemia and microalbuminuria in decreasing frequency. Hypercholesterolemia and low HDL cholesterol were the commonest metabolic abnormalities in the control group.

**Conclusion:**

The prevalence of the MS in persons with T2DM in Nigeria appears to be high. Secondly, there is a high prevalence of lipid abnormalities in all the study groups.

## Introduction

Clustered metabolic cardiovascular risk factors, including type-2 diabetes mellitus, essential hypertension, obesity, dyslipidemia and ischemic heart disease, known as the metabolic syndrome, have been well described [1]. It is estimated that about a quarter of the world’s population have the syndrome and people with this condition are likely to have a increased cardiovascular risk compared with people without it [2]. The risk of developing DM is 5-fold greater in people with this syndrome [3].

In 1988, Reaven [4] described a multifaceted metabolic abnormality consisting of insulin resistance with compensatory hyperinsulinaemia, T2DM, essential hypertension and hypercholesterolaemia. This syndrome became known as Reaven’s syndrome [5]. Today, however, the World Health Organization (WHO) and International Diabetes Federation (IDF) use the term “Metabolic Syndrome” to describe this constellation of conditions [6,7].

It is estimated that about 20-25% of the world’s population have the metabolic syndrome [4]^.^ Its prevalence rates range from 13-30% and 70-80% among the Caucasian nondiabetic [4,5] and diabetic [6,7] populations, respectively. Among African-Americans with T2DM, the prevalence of the metabolic syndrome is estimated to be 70% [8].

Genetically determined insulin resistance in a setting of suitable environmental factors is the pivotal pathogenic mechanism underlying the metabolic syndrome [1]. Interestingly, non-diabetic relatives of diabetics tend to be insulin resistant [9-11]. In addition, both genetic and/or non-genetic familial influences seem to affect the initiation and progression of the metabolic syndrome and also, the concordance rates for glucose intolerance, overall obesity, and low HDL-C were significantly higher among monozygotic than dizygotic twins, indicating a genetic influence on these phenotypes [12]. Other factors known to be associated with MS includes Leptin [13], beta cell dysfunction [14], and insulin resistance [15].

With the metabolic syndrome driving the twin global epidemics of T2DM and cardiovascular disease, there is an overwhelming moral, medical and economic imperative to identify people with this syndrome early so they can benefit from lifestyle interventions and treatment that may alter the course of the disease. The features, causes and consequences of the metabolic syndrome are in a dynamic period of expanding thinking and investigation. Although there are several studies on metabolic syndrome but there is no study that has reported metabolic abnormalities among diabetics and their non-diabetic FDR in Benin City hence the need for this study.

### Aim

To determine the profile of metabolic abnormalities in T2DM persons with metabolic syndrome and their non-diabetic first-degree relatives who also had metabolic syndrome in Benin City.

## Methodology

This was a cross sectional case–control study carried out in the Diabetes Clinic of the University of Benin Teaching Hospital, (UBTH) a 500 bed Federal Government tertiary hospital in Benin City, Edo State in the South-south geopolitical region of Nigeria. The UBTH receives referral cases from Edo State and neighboring States like Delta, Ondo, Ekiti and Kogi States and the Federal Capital Territory, Abuja. A total of 124 subjects were recruited using convenience sampling method from the Diabetes Clinic of the UBTH with the inclusion criteria including people diagnosed as having T2DM presenting to UBTH within the last 24 months using the 1999 WHO criteria [16], people aged 30 years and above, on treatment with oral hypoglycemic drugs plus or minus non-pharmacological therapy and not requiring insulin for survival and finally those who consented to participate for the study. Exclusion criteria included subjects diagnosed of having other types of DM, with T2DM with age < 30 years and who declined being a part of the study. For non-diabetic FDR of persons with type 2 diabetes mellitus, ninety-six subjects were recruited using convenience sampling method from among the out patients department of the UBTH (General practice clinic and consultant out patients clinic) and staff of UBTH and the Inclusion criteria included: Subject must be a first degree relative of a diagnosed T2DM person, should be aged 30 years and above, should not be diagnosed of having diabetes mellitus and finally those who consented to the study while the exclusion criteria includes FDR diagnosed with diabetes mellitus and those that declined being a part of the study.

For control subjects, ninety-six subjects were recruited using convenience sampling method from among the staff of UBTH, and healthy relatives of non-diabetic patients. The inclusion criteria included non diabetic age and sex matched adult with normal fasting blood sugar less than 110 mg/dl while the exclusion criteria included non-diabetics less than 30 years of age, FDR of type 2 diabetic and finally non-diabetics who declined being a part of the study.

### Ethical considerations

Ethical approval was sought from the Ethics and Research Committee of the UBTH before the commencement of the study.

A convenience sampling technique was used to recruit 106 persons with T2DM, 96 people who are first degree relatives of type 2 diabetic persons and 96 controls using a questionnaire administered technique. The following were assessed: anthropometric indices, blood pressure, serum lipid profile, fasting blood sugar, proteinuria, and microalbuminuria [16,17]. The data obtained were analyzed using the statistical software-Statistical package for social sciences [SPSS] version 16. Statistical comparisons were made with student’s t-test for quantitative variables and the Chi-square test was used for comparison of proportions. A p-value of less than 0.05 was taken as statistically significant. The student t-test was used to compare means and test for significant differences in the anthropometric and the metabolic indices.

Three hundred and twenty five persons were enrolled for this study (125 persons living with T2DM, 102 persons who were non-diabetic first degree relatives of T2DM persons and 98 non-diabetic control subjects without a history of DM in a first degree relative). Out of the 125 persons living with T2DM, 124 met the requirements for inclusion into the stage of analysis. The person excluded from the study had incomplete results and an irregularly filled questionnaire. Out of the 102 non-diabetic FDR, 6 persons were dropped on account of incomplete data while for the control group, 2 persons were dropped for the same reason. One hundred and twenty four persons with T2DM, 96 non-diabetic FDR and 96 controls met all the requirements were included in this study. The general total of subjects who participated fully in this study was 316 subjects.

Anthropometric measurements of weight, height, waist circumference and hip circumference were measured for each subject. The weight (Wt) was measured with subjects in light clothing, without shoes using a weighing scale and recorded in kilograms [kg] measured to the nearest 0.1 kg. The height (Ht) was measured, without shoes and the subject standing upright and looking straight ahead (along the coronal plane) using a stadiometer and was recorded to the nearest 0.1 m. The body mass index (BMI) was calculated by the formula: BMI = Weight (kg)/Height^2^(m). The waist circumference (WC) was taken at the point midway between the inferior margin of the rib cage and the iliac crest to the nearest 0.1 cm using the measuring tape. The hip circumference (HC) was measured at the level of the maximal gluteal circumference (along the greater trocanter) to the nearest 0.1 cm with subjects standing erect, hands at the sides and feet together. The waist: hip ratio (WHR) was thereafter determined as the WC divided by the HC. The blood pressure (BP) was measured to the nearest 2 mmHg using a standard mercury sphygmomanometer with subjects in the sitting position and the arms resting on the arms of a chair and the sphygmomanometer at the level of the heart using the 1st and 4th Korotkoff sounds for the systolic and diastolic BP respectively.

#### Laboratory investigations

All subjects were instructed to observe an overnight fast for 8–10 hours before the day of sample collection. About 20 mls of blood was collected from the ante-cubital vein using sterile disposable needles and syringes, for the following investigations;

1. Fasting blood sugar: - 2 mls of blood was collected in fluoride oxalate bottles and then analyzed for plasma glucose within one hour by the glucose oxidase method.

2. Serum lipid profile- Blood was collected in plain bottles, allowed to clot and the serum separated and stored at -20°C until analyzed. The assay was done by enzymatic method using Randox Kit.

3. Urinalysis testing for proteinuria using combi-10 test strips and microalbuminuria using micral test kit. There were two testing protocols: In protocol 1, each subject was ensured to be fever free and free of symptoms of urinary tract infection (dysuria, loin pain and suprapubic pain) prior to urine collection for testing as this can cause a “false proteinuria”. Each urine sample was tested with combi-10 test strips for protein, leukocytes, nitrites and blood. A sample of urine with proteinuria but free of leukocytes, nitrites and blood in the absence of fever was considered as having overt proteinuria. Such a sample was considered as having a “significant proteinuria”. Samples free of protein, leukocytes, nitrites and blood were considered for protocol 2 (test for microalbuminuria). Subjects with history of fever, dysuria and or suprapubic pain and those with leukocytes, nitrites and blood in urine either alone or in whatever combination were investigated and treated for urinary tract infection before being allowed to be involved in the study.

Protocol 2: Samples free of protein, leukocytes, nitrites or blood in protocol 1 above went through this protocol to test for microalbuminuria using micral test strips in subjects without fever, dysuria or suprapubic tenderness. A positive micral test is that with a colour reaction of the test urine with standards provided by the manufacturer.

## Result and discussion

### Demographic characteristics

The mean age (SD) of the 124 persons living with T2DM recruited for the study was found to be 58.6 ± 11.2 years while that of the 96 persons in the control group was 57.69 ± 60.8 years and the mean age for FDR group was 57.4 ± 10.6 years. No significant age and sex differences were observed in these groups and this is suggestive of a study with subjects well matched for age and sex. The mean age of T2DM persons (58.6 ± 11.2 years) is higher than the value reported in Sagamu by Alebiosu and Odusan [18] (53.4 ± 6.3 years) but very close to the mean age of a similar study in Lagos by Ogbera [17] (58.7 ± 9.9 years). These findings further supports the fact that T2DM is a disease of increasing age and that in individuals at high risk for diabetes, increasing age is associated with hyperglycemia and reduced readiness with regard to lifestyle modifications. Thus, age should be considered when planning a lifestyle modification program.

There were more females (59.7%) than males (40.3%) with T2DM in this study. The high proportion of females in this study may be due to the nature of Nigerian women in that more of them seek medical attention than men [16]. This may be different in certain Northern Nigerian communities where purdah is practiced. A similar trend was seen in the study by Ogbera [17] in which out of 973 T2DM persons, 703 (72.3%) were females and 260 (26.7%) were males. The study by Alebiosu and Odusan [18], however, had more males than females. The exact male to female ratio of T2DM persons in Nigeria is not known. Nyenwe et al [19] in Port-Harcourt reported a male: female ratio of 1.4:1 of persons with T2DM.

**Figure 1:**
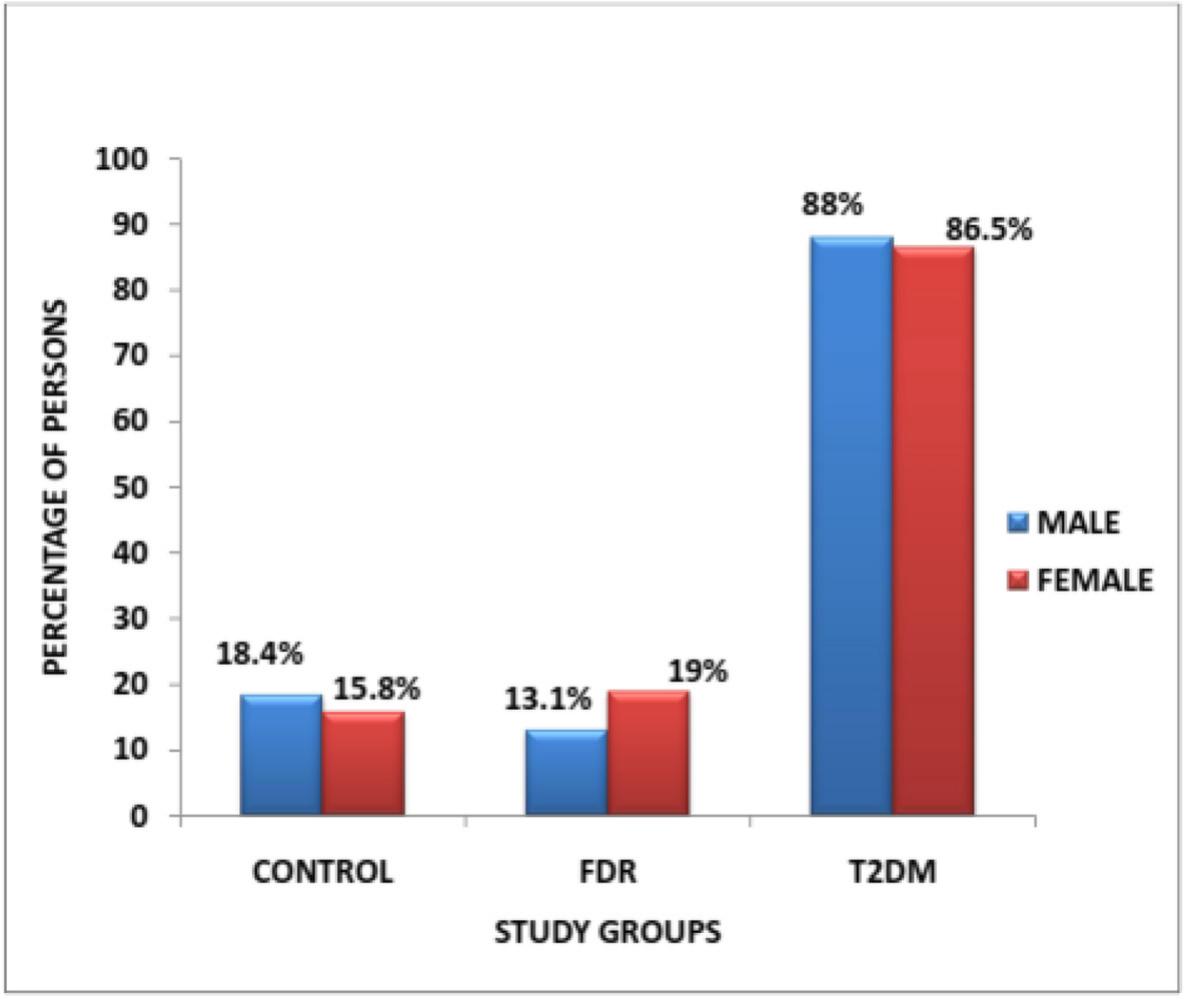
Distribution of persons with metabolic syndrome according to sex.

**Figure 2:**
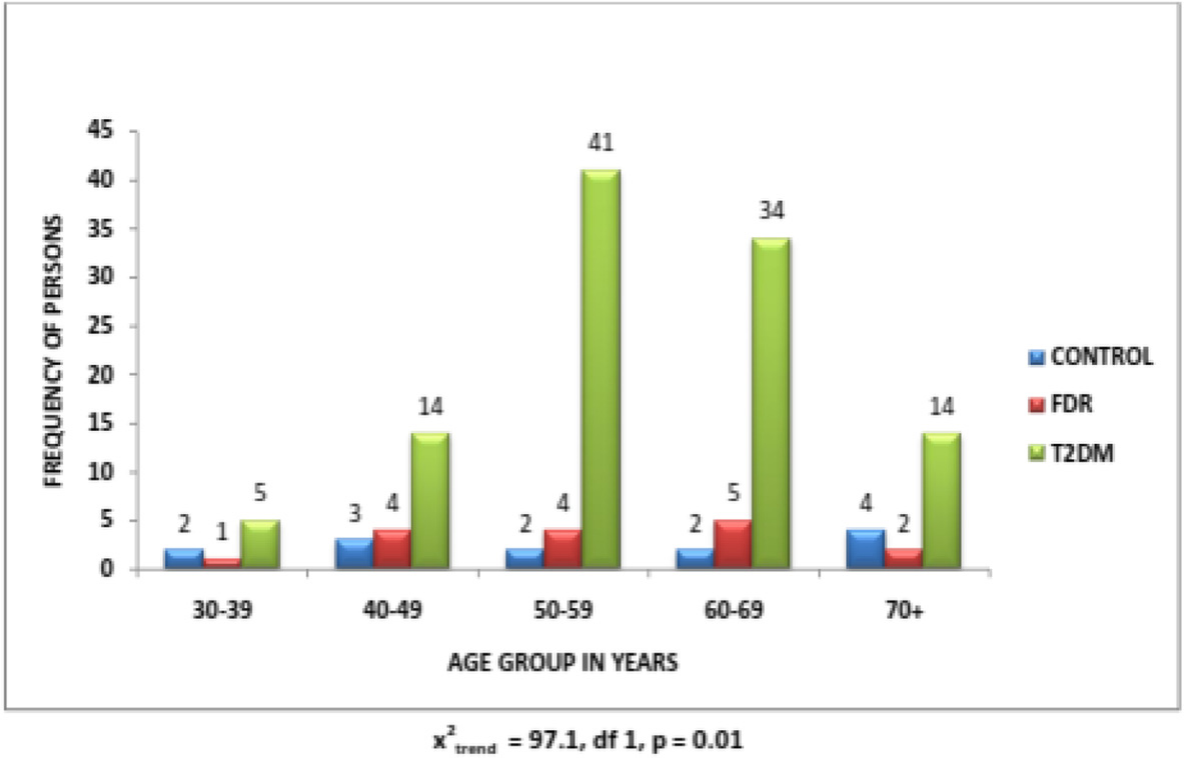
Age distribution of persons with metabolic syndrome in the three study groups.

The prevalence of MS in subjects with T2DM as defined by the WHO criteria (see Table 1) was 87.1% (see Table 2). The sex prevalence of the MS in T2DM males was 88% while that of females was 86.5%. Ogbera [17] reported a prevalence of MS of 86% with a sex prevalence of 86% in females and 83% in males similar to the findings in this study and based on the new streamlined criteria (NCEP-R). The study by Alebiosu and Odusan [18] (which was based on the WHO criteria) reported a prevalence of 25.2% of T2DM patients. This study was, however, concluded in August, 2001. A previous study by Eregie and Edo [20] in 2003 in this locale reported a prevalence of 33.4% in persons with T2DM using the WHO criteria. The work by Adediran [21] in 2003 reported a prevalence of 51.5% in Lagos also using modified WHO criteria in persons with T2DM. In the control group, the general prevalence of MS was 13.5% (13 out of 96 persons). The sex prevalence of males with the MS in the control group was 18.4% (7 out of 38 males) while that of the females was 10.3% (6 out of 58 females). The prevalence of MS in FDR study group was 16.67%. The sex prevalence of MS in males of the FDR group was 13.1% (5 out of 38 males) while that of the female population was of 18.4% (11 out of 58 females). The sex prevalence of MS in the control group was higher in males than females. A similar trend was observed in the T2DM study group. However, in the FDR study group, the sex prevalence was higher in females (see Table 3 and Figure 1).From this study, the frequency of persons with the metabolic syndrome increases with age. For persons with T2DM, the frequency peaked at the age of 50–59 years thereafter, it started declining. The FDR group also showed a similar trend but peaked at the age of 60–69 years before declining at the age range of 70 years and above. The control group also showed a similar trend of increasing frequency with age, however, an early decline was observed. The age range of 50–59 years and thereafter a steady increase with age was observed through to the age of 70 years and above. The chi square for linear trend was 97.1 with a p-value = 0.01 (see Figure 2). This implies there is a linear relationship between age and the metabolic syndrome. Persons with the MS are generally known to have increased risk for cardiovascular events; they are also known to be overweight, hypertensive and to have several metabolic abnormalities. The likely explanation for this is the association of insulin resistance with aging.

**Table 1:** WHO diagnostic guidelines of the metabolic syndrome

	**WHO (1999)**
**Obesity**	**WHR**
	>0.90 (male)
	>0.85 (female)
	or
	**BMI** >30 kg/m^2^
**Serum triglycerides**	≥150 mg/dl
**Serum HDL Cholesterol**	<35 mg/dl (male)
	<39 mg/dl (female)
**Blood Pressure**	≥140/90 mmHg
**Fasting plasma glucose**	**[REQUIREMENT]**
	FPG ≥110 mg/dl
**Other risk factors**	Urinary albumin excretion rate ≥ 20 μg/min or albumin/creatinine ratio
	≥30 mg/g
**Diagnosis**	**Impaired FPG + any 2 criteria**

WHR = waist-hip ratio, BMI = body mass index, FPG = Fasting plasma glucose.

**Table 2:** Prevalence of metabolic syndrome in the 3 study groups using WHO criteria

**Parameters**	**Control**	**FDR**	**T2DM**			
	**n (%)**	**n (%)**	**n (%)**			
	**N = 96**	**N = 96**	**N = 124**	**X**^**2**^	**df**	**p**
**WHO CITERIA**						
**MS**	13 (13.5)	16 (16.7)	108 (87.1)	159.2	2	0.01
**NO MS**	83 (86.5)	80 (83.3)	16 (12.9)			

MS = metabolic syndrome, FDR = first degree relatives, T2DM = type 2 diabetes mellitus.

**Table 3:** Comparison of the demographic parameters of the three groups

**Parameters**	**Control n (%)**	**FDR n (%)**	**T2DM n (%)**			
	**N = 96**	**N = 96**	**N = 124**	**X**^**2**^	**df**	**p**
**Age group**						
30-39 years	7(7.3)	5(5.2)	7(5.6)	2.107	8	0.97
40-49 years	12(12.5)	13(13.5)	15(12.1)			
50-59 years	32(33.3)	35(36.5)	45(36.3)			
60-69 years	28(29.2)	31(32.3)	41(33.1)			
70^+^ year	17(17.7)	12(12.5)	16(12.9)			
**Sex**						
Male	38(39.6)	38(39.6)	50(40.3)	0.017	2	0.99
Famale	58(60.4)	58(60.4)	74(59.7)			
**Level of education**						
No education	6(6.3)	17(17.7)	20(16.1)	15.282	6	0.01^*^
Primary	22(22.9)	24(25.0)	37(29.8)			
Secondary	33(34.4)	16(16.7)	23(18.5)			
Tertiary	35(36.5)	39(40.6)	44(35.5)			
**Marital status**						
Single	4(4.2)	4(4.2)	3(2.4)	8.582	6	0.19
Married	84(87.5)	86(89.6)	112(90.3)			
Divorced	0(0)	0(0)	4(3.2)			
Widowed	8(8.3)	6(6.3)	5(4.0)			

F = Fishers exact test, df = degree of freedom, P = probability value, n = number, N = sample size and * = p < 0.05 (statistically significant).

### Social habits and lifestyle of the study groups

The prevalence of smoking in the control group was 11.5%, 1% in the FDR group and 12.9% in the T2DM group which is higher when compared to other study groups. Several studies [22,23] have identified smoking as an independent risk factor for T2DM. The percentage of study subjects who indulged in alcohol consumption was highest in patients with T2DM (40.3%) followed by the control group (30.2%) and FDR (8.3%) and this was statistically significant. This finding is in keeping with the outcome of a study by Puepet and Ohwovoriole [24] in Jos, Nigeria on the prevalence of risk factors for diabetes mellitus in a non-diabetic population which reported that alcohol consumption appears to be one of the dominant risk factors for development of type 2 DM in this group of upland Nigerians. This was also similar to findings in a study by Lombrail et al. [25] in Paris, France to investigate the link between alcohol consumption and glycoregulation/diabetes mellitus which reported that alcohol consumption was independently associated with diabetes mellitus.

About 60.4% of the control group and 45.8% of the FDR group were not active (using the leisure activity grading) compared to 45.2% of the T2DM group. This finding was in keeping with the outcome of a study done by Helmrich et al. [26] which examined patterns of physical activity and other personal characteristics in relation to the subsequent development of NIDDM in 5990 male alumni of the University of Pennsylvania. They reported that increased physical activity is effective in preventing NIDDM, and the protective benefit is especially pronounced in persons at the highest risk for the disease. It was interesting to note that the T2DM group had the lowest rate of physical inactivity. This may be attributed to their possible adherence to lifestyle modification advice (vis-a-vis exercise) given routinely during follow-up visits to the Diabetes Clinic and at Diabetes association meetings and seminars. (See Table 4).

**Table 4:** Shows a comparison of the social habits and lifestyles of the groups

**Parameters**	**Control n (%)**	**FDR n (%)**	**T2DM n (%)**			
	**N = 96**	**N = 96**	**N = 124**	**X**^**2**^	**df**	**p**
**Smoking**						
Yes	11(11.5)	1(1.0)	16(12.9)	10.579	2	0.01*
No	85(88.5)	95(99.0)	108(87.1)			
**Alcohol use**						
Yes	29(30.2)	8(8.3)	50(40.3)	28.247	2	0.01*
No	67(69.8)	88(91.7)	74(59.7)			
**Work related physical activity grade**						
Not active (sedentary)	43(44.8)	47(49.0)	52(41.9)	1.285	4	0.86
Moderately active	48(50)	43(44.8)	64(51.6)			
Very active	5(5.2)	6(6.3)	8(6.5)			
**Leisure activity grade**						
Not active	58(60.4)	44(45.8)	56(45.2)	7.928	4	0.09
Moderate active	24(25.0)	37(38.5)	52(41.9)			
Very active	14(14.6)	15(15.6)	16(12.9)			

X2 = chi square, df = degree of freedom, p = probability value, n = number, N = sample size and * = p < 0.05(statistically significant).

### Comparison of biochemical variables between the control and T2DM group

The mean FBS (117.2 ± 5.7 mg/dl) of the control group was lower than that of the T2DM group (139.7 ± 17.1 mg/dl). However, the mean total cholesterol and triglycerides were higher in the control group than in the T2DM group. This may be a reflection of the unrestricted diet in the control group and a reflection of therapy in persons with T2DM. A few of the control subjects were on therapy for hypertension and the drug (thiazide) effect may reflect on their mean blood glucose. The mean values of FBS, total cholesterol, HDL cholesterol and LDL cholesterol were higher in the persons with the MS in the T2DM group than in persons with the MS in the FDR group. These findings were, however, not significant. The mean values of triglyceride were higher in persons with the MS in the FDR group than in persons with the MS in the T2DM group (see Table 5).

**Table 5:** Biochemical variables of persons with the metabolic syndrome in the control and type two diabetes mellitus groups

	**Control with MS**	**T2DM with MS**			
	**n = 13**	**n = 108**			
**Parameters**	**Mean ± SD**	**Mean ± SD**	**t**	**df**	**P**
**AGE(years)**	55.6 ± 16.0	57.4 ± 10.4	0.54	119	0.59
**FBS(mg/dl)**	117.7 ± 5.6	139.7 ± 60.3	1.298	119	0.19
**Total cholesterol(mg/dl)**	190.9 ± 57.6	184.9 ± 47.9	0.358	119	0.68
**HDL cholesterol (mg/dl)**	60.7 ± 32.4	49.0 ± 15.8	2.180	119	0.03*
**LDL cholesterol (mg/dl)**	95.1 ± 48.1	107.2 ± 33.97	0.854	119	0.22
**Triglycerides(mg/dl)**	172.2 ± 48.1	117.2 ± 45.8	2.537	119	0.01*

FBS = Fasting blood sugar, HDL = High density lipoprotein, LDL = Low density lipoprotein, MS = Metabolic syndrome and SBP = systolic blood pressure.

### Comparison of biochemical variables between the FDR and control group

Comparing persons with the MS in the FDR group with that of persons with the MS in the control group, the persons with MS in the FDR group tended to be more overweight, with more central obesity than persons with the MS in the control group. The BMI was found to be significantly different. This may imply an inherent familial tendency of being overweight or obese with a resultant development of DM in some family members. Persons in the FDR group had higher mean systolic and diastolic blood pressures which may be a reflection of their degree of being overweight or obese. The persons in the control group with the MS had a slightly lower mean FBS (117.2 ± 5.7 mg/dl) than persons in the FDR group with the MS (118.4 ± 6.1 mg/dl). Although the difference was not significant, this was not unexpected as previous studies have demonstrated that the first degree relatives of Type 2 diabetic patients constitute a high risk group for DM [27]. Another study by Ma H et al. [28] on the prevalence of diabetes and prediabetes mellitus in the first-degree relatives (FDR) of patients with type 2 diabetes (T2DM) in Chengdu, China reported high risk of diabetes in FDR of T2DM patients. The mean total cholesterol and triglycerides were higher in persons with the MS in the control group than that of persons with the MS in the FDR group. This may be as a result of dietary adjustments of the FDR persons because of their potential risk for developing DM. These findings were, however, not significant (See Table 6).

**Table 6:** A comparison of biochemical indices of persons with the metabolic syndrome in the control group and the group of first degree relatives of persons living with type two diabetes mellitus

**Parameters**	**Control with MS**	**FDR with MS**			
	**n = 13**	**n = 16**			
	**Mean ± SD**	**Mean ± SD**	**t**	**df**	**p**
**Age (years)**	55.6 ± 16.0	54.4 ± 11.8	0.228	27	0.82
**FBS (mg/dl)**	117.7 ± 5.6	118.4 ± 6.1	0.335	27	0.74
**Total cholesterol (mg/dl)**	190.9 ± 57.6	171.1 ± 55.6	0.935	27	0.35
**HDL cholesterol (mg/dl)**	60.7 ± 32.4	48.6 ± 15.8	1.264	27	
**LDL cholesterol (mg/dl)**	95.1 ± 48.1	99.3 ± 44.9	0.242	27	0.81
**Triglycerides (mg/dl)**	172.2 ± 48.1	155.6 ± 34.3	0.763	27	0.48

FBS = Fasting blood sugar, HDL = High density lipoprotein, LDL = Low density lipoprotein, MS = Metabolic syndrome and SBP = systolic blood pressure.

### Comparison of biochemical features of persons with MS in the T2DM and FDR group

The comparison of the clinical and biochemical features of persons with the MS in the T2DM and the FDR groups revealed that though persons with the MS in the T2DM group had higher mean of WC, FBS, total cholesterol, HDL, LDL and triglycerides compared to the FDR subjects with the MS, this was not significant (See Table 7).

**Table 7:** Comparison of biochemical variables of persons with the metabolic syndrome in the type two diabetes mellitus group and the group of first degree relatives of persons living with type two diabetes mellitus

	**T2DM with MS**	**FDR with MS**			
	**n = 108**	**n = 16**			
**Parameters**	**Mean ± SD**	**Mean ± SD**	**t**	**df**	**P**
**Age (years)**	57.4 ± 10.4	54.4 ± 11.8	1.037	122	0.30
**FBS (mg/dl)**	139.7 ± 60.3	118.4 ± 6.1	1.391	122	0.16
**Total cholesterol (mg/dl)**	184.9 ± 47.9	171.1 ± 55.6	1.058	122	0.29
**HDL cholesterol (mg/dl)**	49.0 ± 15.8	48.6 ± 15.8	0.099	122	0.92
**LDL cholesterol (mg/dl)**	107.2 ± 33.9	99.3 ± 44.9	1.463	122	0.14
**Triglycerides (mg/dl)**	117.2 ± 45.8	155.6 ± 34.3	1.782	122	0.07

FBS = Fasting blood sugar, HDL = High density lipoprotein, LDL = Low density lipoprotein, MS = Metabolic syndrome and SBP = systolic blood pressure.

### P**rofile of the metabolic abnormalities in the three study groups**

**Figure 3:**
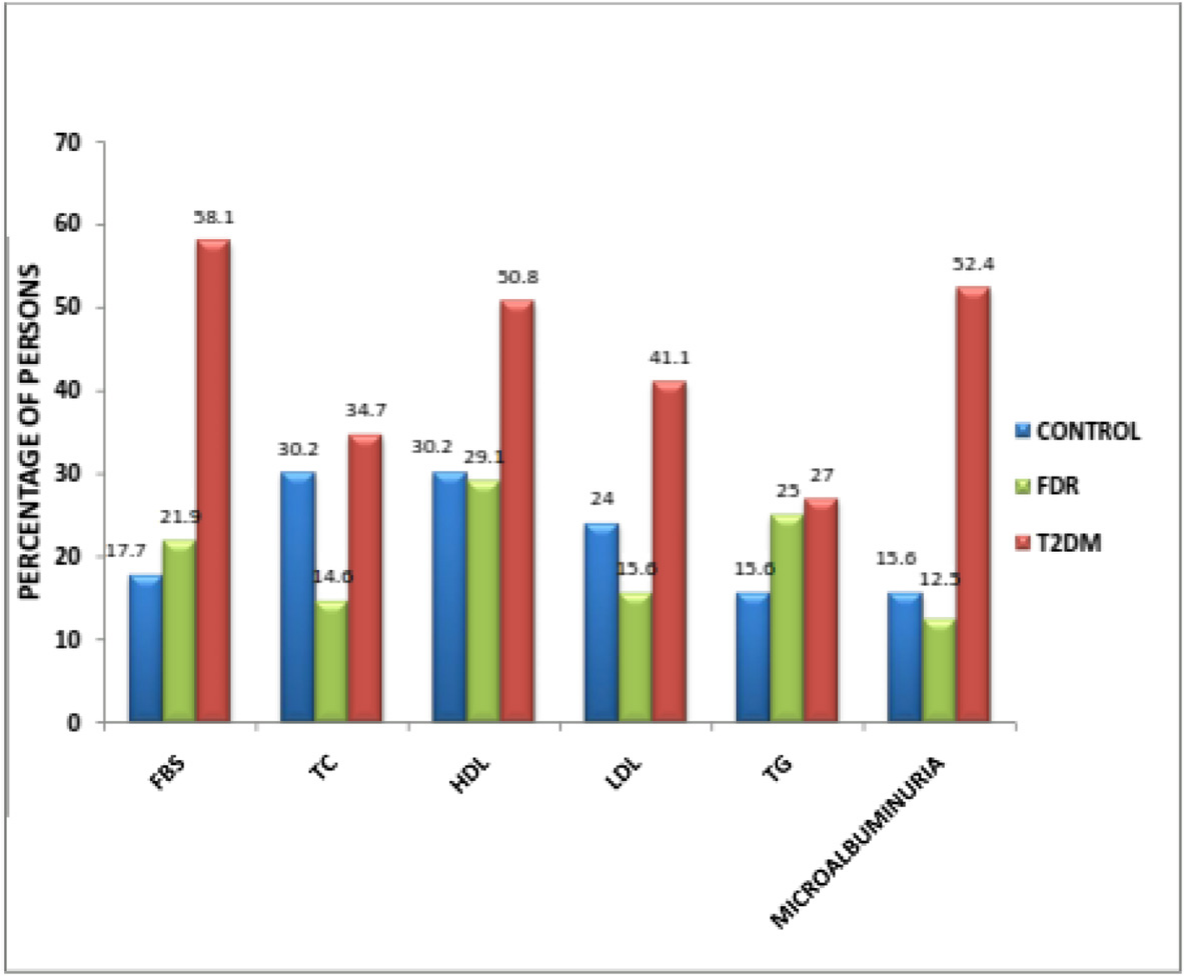
Profile of metabolic abnormalities in the three study groups.

of the 38 males in the control group, 21.0% had impaired fasting blood glucose, 36.8% had hypercholesterolaemia, 7.9% had low HDL cholesterol, 5.2% had elevated LDL cholesterol and 10.5% had hypertriglyceridaemia. The female in this group, however, had higher frequency of biochemical abnormalities. Of the 58 females in this group, 15.5% had impaired fasting blood glucose, 25.9% had hypercholesterolaemia, and 20.0% had low HDL cholesterol level. Of the 38 males in the FDR group, 18.4% had impaired fasting blood glucose, 10.5% had hypercholesterolaemia, 10.5% had low HDL cholesterol, 7.9% had elevated LDL cholesterol and 47.4% had hypertriglyceridaemia. Of the 58 female in this group, 24.1% had impaired fasting blood glucose, 7.2% had hypercholesterolaemia, 6.9% had low HDL cholesterol level, 6.8% had elevated LDL and 6.8% had hypertriglyceridaemia (see Figure 3).

The mean values of TC, HDL and TG were lower in the FDR of persons with T2DM. When compared to the control group, persons in the FDR group had lower means than those in the control group. This may be a reflection of lifestyle adjustments they have made knowing their inherent risk of developing T2DM in future. Such lifestyle adjustments may involve the avoidance of refined sugars, reduced smoking and alcohol ingestion and increased physical activities. Though persons in the FDR group had a higher tendency of being overweight than the persons in the control group, the smoking habit, alcohol ingestion and use of herbal medications (which may be alcohol based) were found to be significantly higher in persons in the control group and these may also explain the higher mean values of FBS, TC, LDL and TG observed in them. Low HDL is the commonest metabolic abnormality in the FDR group, followed by hypertriglyceridaemia, impaired fasting glucose, high LDL, hypercholesterolaemia and microalbuminuria respectively. The mean values of the major metabolic abnormalities in persons with T2DM were found to be relatively higher than that of persons in the control group. Similarly, the T2DM group had higher mean biochemical indices than that of the FDR group. Impaired fasting glycaemia is the commonest metabolic abnormality in persons with T2DM followed by microalbuminuria, low HDL, elevated LDL, hypercholesterolaemia, and Hypertriglyceridaemia respectively. These findings suggest persons with T2DM are a risk group we should routinely investigate periodically.

## Conclusion

The prevalence of the metabolic syndrome in Nigerians with T2DM is high compared to that of control and non-diabetic first degree relatives. Abnormal glucose reading is the commonest metabolic abnormality in persons with T2DM followed by microalbuminuria, low HDL, elevated LDL, hypercholesterolaemia, and Hypertriglyceridaemia respectively. Low HDL is the commonest metabolic abnormality in the FDR group, followed by hypertriglyceridaemia, impaired fasting glucose, high LDL, and hypercholesterolaemia respectively while impaired fasting glucose was noted in both the control and FDR. Poor glycaemic control (FBS) and Hypertriglyceridaemia are significant biochemical abnormalities in T2DM persons with the MS. There is need for concerted efforts by family members, communities, healthcare professionals and patient based groups towards preventive based approach in the management of diabetes and metabolic syndrome; this can be achieved through increased emphasis on lifestyle modification strategies such as exercise, increased dietary restrictions and weight control strategies especially for those with impaired fasting glucose. A major strength of this study is that it was the first study in Benin City that looked at metabolic abnormalities in T2DM and their FDR with metabolic syndrome: to the best of our knowledge, there has not been any previous study on this interesting group of subjects. Limitations that arose from this study includes: 1) This was a small study therefore a larger and specifically designed study is needed to evaluate the metabolic abnormalities in T2DM patients, their FDR and cohorts, 2) We did not measure HbA1c levels in this study.

## Competing interest

The authors of this article declare that they have no competing interests.

## Authors’ contributions

OS have made substantial contributions to conception and design, acquisition of data, analysis and interpretation of data; EI have been involved in drafting the manuscript and revising it critically for important intellectual content. Both authors read and approved the final manuscript.

## Authors’ information

OS (MBBS, FMCP) is a consultant Physician/Endocrinologist, member; Nigerian Society of Endocrinologist and Metabolism (NSEM), American Association of Clinical Endocrinologist (AACE) and a practising Endocrinologist, has research interest in metabolism and obesity. EI (MBBS, FMCP) is a consultant Physician/Endocrinologist, member; Nigerian Society of Endocrinologist and Metabolism (NSEM), American Association of Clinical Endocrinologist (AACE) and has research interest in Diabetic emergencies.
